# Consequences of M&A team composition for deal outcomes: An inductive study

**DOI:** 10.3389/fpsyg.2022.931025

**Published:** 2022-10-03

**Authors:** Timo Paumen, David Kroon, Svetlana N. Khapova

**Affiliations:** Department of Management and Organization, Vrije Universiteit Amsterdam, Amsterdam, Netherlands

**Keywords:** M&A, M&A teams, M&A learning, M&A team culture, M&A success

## Abstract

While Merger & Acquisition (M&A) activity has reached unprecedented levels over recent years, M&A failure rates remain high. In explaining these disappointing outcomes, previous studies barely focused on the teams that manage these M&A transactions. Furthermore, only scant information exists on team members’ roles and skill sets. With an aim to contribute to filling this gap, we inductively explore a composition logic of M&A teams and its consequences for M&A outcomes by following a grounded theory approach and conducting semi-structured interviews with 30 M&A professionals. We identify three prevailing team roles (project manager, expert and executor) which require a specific set of soft and hard skills that explain how M&A teams can enable M&A success in terms of deal execution and completion. Furthermore, we provide evidence to how aspects of project timing, such as deadlines and simultaneous projects, shape the team members’ work. Finally, our findings underline the importance of accumulating experience and learning effects on M&A deal outcomes, shaping both M&A team culture and the team members’ skill sets.

## Introduction

Merger and acquisition (M&A) activity has reached unprecedented highs throughout the last decade (Meynerts-Stiller and Rohloff, 2019a). Despite the significant increase in the amount of transactions closed, many of them continue to fail over time (Jiaxin, 2021). Unsurprisingly, both scholars and practitioners spent a lot of time and budget on researching how M&A deal outcomes could be more successful (Renneboog and Vansteenkiste, 2019). As such, various perspectives on M&A deal outcomes have been offered over the past decades (Haleblian et al., 2009). What is striking, however, is that little research attention has been given to the role of a team that manages M&A transactions (Aktas et al., 2021). This focus is, however, highly important considering the effects of M&A teams and their composition on the M&A deal success (Aktas et al., 2021). Specifically, research shows that M&A teams have an important influence on the rationales behind deal making and on the screening of potential takeover targets (Aktas et al., 2021). For example, teams with strong financial expertise are found to be more prudent in their evaluation techniques and to exhibit a higher level of diligence, thus increasing negotiations time (Aktas et al., 2021; Graham and Harvey, 2001). In turn, teams with higher managerial overconfidence often act faster, but could be more prompt to decision errors (Malmendier and Tate, 2008). Research further suggests that there is an effect of descriptive team characteristics on M&A deal outcomes, such as gender diversity (Adams and Ferreira, 2009), nationality diversity (Nielsen and Nielsen, 2013), and team tenure (Aktas et al., 2013). Hence, although there a number of studies addressing teams that manage M&A transactions, little has been done to study this topic more in-depth, particularly not from a deal making process perspective.

With this paper we aim to contribute to filling this gap. We focus on exploring a composition logic of M&A teams and its consequences for M&A outcomes by answering the following research question: *How do M&A team compositions shape M&A deal outcomes?*

To conduct our study, we applied a qualitative research design and conducted semi-structured interviews with 30 M&A professionals. In contrast to quantitative research, qualitative studies offer the opportunity to explore vastly new concepts (Goulding, 2002). We followed a grounded theory approach and analyzed the interview transcripts adhering to the ‘Gioia methodology’ (Gioia et al., 2013). Our methodological approach included the development of a theoretical framework which emerged from our understanding of the relationships between the identified concepts.

Our findings illustrate the existence of three team roles (i.e., the project manager, expert and executor). These roles entail different sets of tasks and responsibilities which all have the same goal of working towards the M&A deal completion. While the project manager is primarily responsible for task delegation and stakeholder management, the experts add value through their knowledge on financial and commercial matters. The executors support the project managers and experts with fulfilling their tasks. The combination of the identified roles and subsequent clear allocation of responsibilities in M&A teams further drive a smooth execution of an M&A transaction. We also present four soft skills which are beneficial to the M&A deal completion and execution, especially when captured in project managers. These soft skills are comprised of communication, relationship management, negotiation and emotional intelligence. We finally identify and elaborate on two hard skills, particularly in the context of the expert role (i.e., industry knowledge and financial expertise).

By developing an empirically-grounded model of how M&A teams shape deal outcomes, we reveal a learning cycle which explains how team members work towards M&A deal completion. The more deals are completed, the more refined the skill set becomes, which consequently prepares the individual team roles to perform better with regard to the next deal. Besides addressing team composition, our findings present novel insights on the M&A team level by illustrating how tight deadlines, different M&A phases and simultaneous transactions affect the relationship between M&A team composition and M&A deal execution. Finally, we elucidate the role of a team culture which is shaped by shared language, trust and relationships.

Our study answers the call for more research on M&A team characteristics in relation to M&A performance (Aktas et al., 2021). Hence, by adopting a team perspective, we try to provide more insight into the M&A process and its outcomes. Furthermore, we contribute to research on M&A learning where findings have been contradicting so far. For example, studies on managerial overconfidence state that learning triggers top executives to develop the so-called confirmation bias which induces worse decision-making and eventually value destruction (Garbuio et al., 2010). However, other scholars found positive effects of learning in an organizational context with regard to, e.g., post-merger integration (Barkema and Schijven, 2008; Collins et al., 2009). Aside these positive and negative effects, some scholars argue that learning hardly happens in the M&A context, as these transactions are so unique and not repetitive (Bower, 2004). We enrich this current body of knowledge by adding insights on positive learning effects at the individual level.

From a practical perspective, we offer suggestions on how M&A departments in corporations, as well as consulting firms or investment banks, can apply the insights on how role allocation and respective skill sets are deployed in successful M&A teams. Furthermore, our insights on project timing and team culture could help improve team members working towards positive M&A deal outcomes.

The remainder of this paper is structured as follows. First, we present the theoretical background of this study and elaborate on the research gap we found. Following this, we lay out our methodology. Then, we present our findings and develop a theoretical model of how M&A teams affect deal outcomes. Finally, we discuss what our findings imply for the existing M&A literature and provide future research directions.

## Theoretical background

### M&A teams

Higgins et al. (2012) argue that findings on team composition largely vary in terms of the context in which a team is operating. While the current state of team literature offers quite some insights on the role of teams in sports and entrepreneurship (Steinbrink et al., 2020), we do not know much about M&A teams and their role in explaining M&A success. However, M&A teams can be seen as crucial and may have a lot of responsibility as they are executing the M&A transactions (Nadolska and Barkema, 2014). Failure here could not only induce shareholder value destruction but also employee dissatisfaction.

The tasks of M&A teams, including firm-internal employees as well as external advisors, are largely related to information collection and data analysis, as well as reporting to CEOs and members of a firm’s board (Kang et al., 2020). The right selection of project managers in these M&A team is of great importance (Kajackaite and Sliwka, 2020). However, there are relatively few studies focusing on project manager selection (Šiško Kuliš, 2020). We do know that required competencies include tacit and explicit knowledge and skills dealing with complexity and financial risk, which can be captured under “hard” and “soft” skills (Šiško Kuliš, 2020).

Hard skills are defined by a certain type of expertise which can easily be documented and attained (Sopa et al., 2020). In addition, it can be created, written and even transferred between teams or groups in companies (Lombardi, 2019). In other contexts, scholars have focused on hard skills, such as obtaining technical knowledge (Edum-Fotwe and McCaffer, 2000; Odusami, 2002). Soft skills, on the other hand, are a type of knowledge connected to the human mind and personality. They are difficult to formulate and transfer to others (Holford, 2019). Furthermore, soft skills are based on actions and experiences, which involves values and emotions (Asbari et al., 2020). Studies on soft skills in an M&A context have focused on stress resilience and performing well under pressure (Meynerts-Stiller and Rohloff, 2019b), as well as teamwork (Brand, 2020). Brand (2020) identifies the ability to work in a team as crucial in M&A projects. Due to the regular time pressure in completing M&A transactions, team members need to rely on one another.

Team structure and team roles also become important when teamwork is omnipresent in complex and dynamic environments such as in the M&A context. Uncertainty, high-risk characteristics and high tempo call for efficient teamwork (Brehmer, 2007). Team roles are defined as a set of instructions determining how a team member should behave (Biddle and Thomas, 1986). Role allocation is influenced by the expertise of different team members which the team needs to coordinate in order to achieve their goal (Jobidon et al., 2017). The interplay between so-called project-related roles plays an important role in team performance as well (Allen et al., 1988).

Higgins et al. (2012) further suggest that role stability has a positive impact on team performance, especially in the context of organizational change. In line with this, Jobidon et al. (2017) argue that role variability can be beneficial only in very specific contexts, such as self-organized groups. In addition, Savelsbergh et al. (2015) found evidence that team role stability increases learning effects among all team members which could be beneficial in an M&A context. This is important, as M&As bear the risk of failure and learning effects might mitigate this risk. The following sub-chapter will delve deeper into M&A learning.

### M&A learning

Professionals gather M&A experience and learn from previous deals (Collins et al., 2009). Trichterborn et al. (2016) describe M&A learning as refining articulation, sharing and internalization among involved parties. In the M&A literature, findings on learning are contradicting due to the multi-facetedness of M&A success (Zollo and Meier, 2008). First, literature on managerial hubris points out that learning makes executives develop the so-called confirmation bias which induces worse decision-making and shareholder value destruction (Billett and Qian, 2008; Garbuio et al., 2010). Second, Zollo (2009) observes that at the individual level the managers’ perception of the M&A deal outcome increasingly deviates from objective performance measures when accumulating deal experience. At the organizational level, Zollo and Singh (2004) conclude that firms with more post-merger integration experience do not significantly integrate better. On the other hand, multiple studies found positive effects of M&A learning (Collins et al., 2009). Trichterborn et al. (2016), for example, present evidence that establishing an M&A function within an organization, which only purpose is the management of M&A deals, has a positive effect on M&A performance. They further argue that it is necessary for firms to capture prior experience and to accumulate knowledge related to the M&A process and management know-how. In line with this, Kengelbach et al. (2012) illustrate that capturing deal experience and building M&A know-how is not just important for the deal phase but also for the post-merger integration process, especially due to the complexity of soft and hard integration decisions (Kroon et al., 2022).

In sum, as the role of team composition, roles and learning with regard to M&A deal outcomes has not been researched in-depth, despite its importance, we want to contribute to filling this research gap by raising the following research question: *How do M&A team compositions shape M&A deal outcomes?* In the following chapter, the current paper’s underlying methodology will be presented.

## Materials and methods

### Research design

Our objective is to explain the role of M&A teams with regard to M&A deal outcomes and to extend existing theory “by making it more dense by filling what has been left out – that is by extending and refining its existing categories and relationships” (Pratt et al., 2006, p. 238). As we aim to generate and discover new theory, we opted for grounded theory as a methodology (Glaser and Strauss, 2017). This approach is particularly useful for research to predict and explain behavior or perceptions of individuals with the goal of advancing and creating theory (Goulding, 2002). Following an interpretive research philosophy and theory development approach, we chose a qualitative research design.

### Research context and data collection

A focus upon details and the reality behind these details leads us to a data collection technique including small samples and in-depth investigations (Basias and Pollalis, 2018). This approach adheres to an inductive theory development procedure. In-depth interviews with 30 M&A professionals were held in order to explain, better understand, and explore the research subjects’ opinions and experiences (see Table 1 for sample descriptions). Because of the research question’s exploratory nature, these interviews have a semi-structured design (Easterby-Smith et al., 2008). An interview protocol was created in order to have a list of themes and questions covered, although some of them varied among the interview sessions (see Appendix I). Thus, flexibility was granted with regard to the individual interviewee’s context and the participants were free to deviate from the interview protocol in the direction they felt to be important. Moreover, additional questions may have been added if a newly introduced concept was initially rather superficially discussed by the interviewee.

**Table 1:** Sample descriptives.

Interviewee	Gender	Tenure (in years)	Firm	Position/Function
1	female	3	A	Assistant Manager
2	male	3	B	Associate
3	male	3	C	M&A Associate
4	male	2	D	Consultant
5	male	5	A	Assistant Manager
6	male	26	A	Senior Manager
7	male	5	A	Assistant Manager
8	female	3	A	Assistant Manager
9	male	3	E	Associate Consultant
10	male	7	F	Director
11	male	17	A	Manager
12	male	8	A	Senior Manager
13	male	3	G	M&A Analyst
14	male	15	A	Senior Manager
15	male	5	A	Assistant Manager
16	male	12	A	Senior Manager
17	female	2	H	Associate
18	male	9	F	Associate Director
19	male	5	I	M&A Analyst
20	male	2	A	Senior Associate
21	male	3	D	Consultant
22	male	2	D	Consultant
23	male	2	A	Senior Associate
24	male	11	A	Manager
25	male	25	A	Partner
26	female	6	A	Assistant Manager
27	female	3	A	Assistant Manager
28	female	5	A	Manager
29	male	7	F	Associate Director
30	male	9	A	Senior Manager

The interviewees were provided with an information letter and informed consent describing the study’s aim and context which assures anonymization and therefore reduces the pressure to give answers that are socially acceptable. The interviews were held via the video communication platform Zoom and lasted on average 45 min. The interviews were structured as follows. In the beginning, small talk was held in order for the participant to feel comfortable, to ask his/her remaining questions prior to the start and to establish a trustful relationship. Then, the interviewee was informed that the recording would begin. After having the official interview, the recording was stopped, and a short debriefing was done. Finally, the interview recordings were manually transcribed using the software Express Scribe. The final word count of the full data set amounts to 124,584 words which averages 4,152 words per interview transcript.

### Data analysis

Our data analysis approach complies with existing standards set by Gioia et al. (2013), known for their detailed and explicated procedures of coding. We analyzed the interview transcripts in an iterative and circular approach going back and forth between the data and the developing pattern of theoretical categories (Corbin and Strauss, 1990; Strauss and Corbin, 1998). The executed analysis was segmented in four steps:

*Step 1: Creating provisional categories and first-order codes.* The interview transcripts were inserted in the coding software Atlas.ti. Here, we started open coding the interviewee’s answers and continuously identified provisional categories and first-order codes. The coding was done by two researchers of the research team which yielded a very high agreement (> 90%). In bi-weekly calls any deviating codes were discussed and aligned. As already mentioned, this being an iterative process, first-order codes were re-named and slightly adjusted throughout the analysis of more and more transcripts. After codes have been reviewed again, 37 first-order codes emerged from open-coding all interviews. Examples of such codes are “clear definition of responsibilities” and “coordination problems because of language.”

*Step 2: Consolidation of first-order codes and creating second-order themes.* In a second step, we consolidated and merged some first-order codes which were initially named differently but described the same phenomenon, such as “project head” and “project manager.” This resulted in a reduction of 4 first-order codes. By merging some of the concepts, they became more theoretical and abstract. Thus, we moved from open to axial coding (Strauss and Corbin, 1998; Gioia et al., 2013).

*Step 3: Aggregation of theoretical dimensions.* After second-order categories were identified, we further clustered them in order to generate an abstract understanding. Selective coding was applied in order to generate theoretical dimensions which encompassed the second-order categories, e.g., “M&A team culture” was formed of “shared language,” “trust,” and “relationships.”

**Figure 1:**
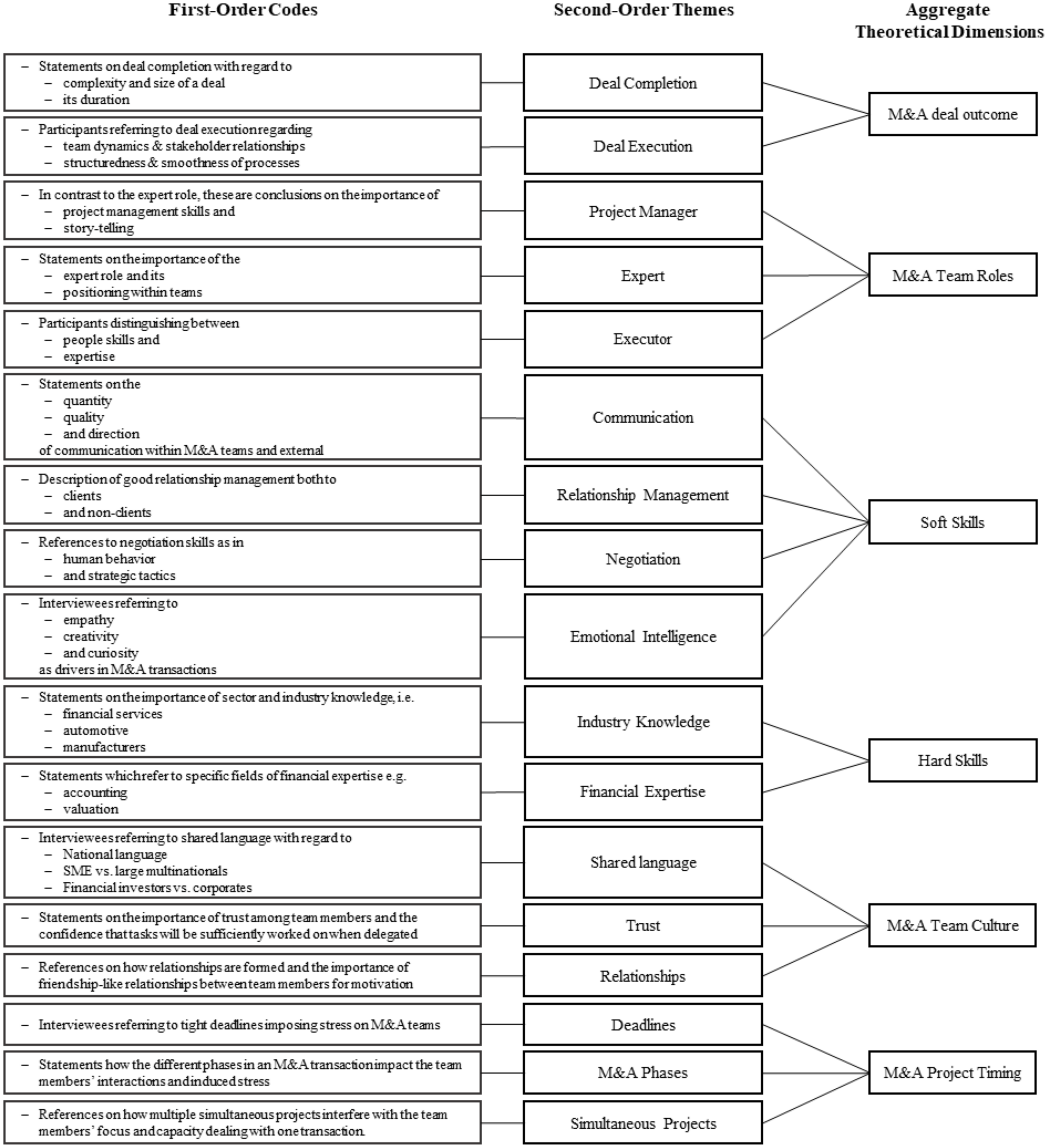
Data structure.

Figure 1 illustrates the data structure that we developed. It describes the identified first-order codes, second-order themes and theoretical dimensions derived from analyzing the interviews held.

*Step 4: Developing theoretical framework.* Lastly, our methodical approach included the development of a theoretical framework which emerged from our understanding of the relationships between the theoretical dimensions and second-order categories (Gioia et al., 2013). Based on the data structure and relevant quotes within the data, in combination with existing literature, we were able to develop an empirically-grounded model.

### Trustworthiness of the study

We applied certain measures in order to enhance the trustworthiness of our study, in particular the credibility, dependability, transferability and confirmability of this paper’s underlying research (Lincoln and Guba, 1985). First, we checked whether findings of both junior and senior professionals deviated. No material inconsistencies were found which strengthens the credibility of the findings (Patton, 2015). Second, to ensure the authenticity and correct understanding of the interviewees’ responses, we summarized answers during the interviews and asked for the respondent’s confirmation and validation, as suggested by Johnson and Waterfield (2004). In order to increase the transferability of our findings, we provided rich detail of context and thick descriptive data. Finally, we outlined the entire research process as transparently as possible in order to increase dependability and confirmability. Especially with regard to coding, we enhanced dependability by jointly discussing unclear concepts (Noble and Smith, 2015).

## Findings

As we conducted our data analysis, critical themes began to emerge. In the following sections, we provide an overview of these themes. We begin by introducing the concept of M&A deal outcome. Following this, we will present different project-related team roles and explain which soft and hard skills within M&A teams drive deal outcomes. Moreover, we present findings on M&A project timing, as well as team culture and theorize how these themes influence the relationship between M&A team composition and M&A deal outcomes. This results in an empirically-grounded model of how M&A team roles and skills shape perceptions of M&A deal outcomes. In Table 2 further quotes are presented to provide additional evidence to our findings.

**Table 2:** Illustrative evidence - interview quotes.

ID	Quote
Deal Completion	
3:1	The most successful transaction would have been Project Arthur because it was the largest project.
5:1	In the first moment, I think of the two largest transactions.
2:2	The deal went through quickly.
Deal Execution	
7:4	I partly had the feeling that what we were doing was not really well structured. It was not clear to everyone what had to be done and what had to be done in what timeframe.
28:8	There are very different points, so I think on the one hand, if you already know the customer for a long time and have delivered good work there in the past, it is actually so that the trust or this level of trust is built up due to the past work performance and thus you have a good customer loyalty. When you first contact a customer, I think it’s important to listen to the customer, to take on board his points and to try to make him understand and take on board his issues. In other words, you have to give them the feeling that their issues are being addressed and evaluated appropriately.
6:1	That was the measure of success, that it was a good service for the customer.
7:35	With our DD projects, I sometimes have the feeling that we are doing this for the first time. I think it’s also a bit of a problem that the projects are managed by colleagues who do not have that much experience and are still very excited themselves. What you need to make this better are experienced people who approach it calmly and carry it out in a well-structured way.
Project Manager	
27:13	The project manager, who for me usually has more of a PMO role, is certainly very important. He may not necessarily have in-depth technical knowledge, but he has an overview of the entire project. Especially when there are many different workstreams, many different interest groups, who keeps them together and drives the whole project forward and makes sure that deadlines are met.
24:16	In principle, the project manager then does this at the operational level and, in particular, also leads the entire sub-teams accordingly. At the interpersonal level, the project coordinator naturally needs a certain amount of experience in order to have an overall understanding of the complexity of transactions on the one hand. As a rule, I no longer expect the project coordinator to be the absolute expert on all the details, but rather to have an overall strategic understanding of a transaction, and on the other hand, he or she must be very communicative, because he or she must maintain constant communication with the client in particular, and ultimately he or she must also demand rapid work results from the sub-teams in a high-stress situation and then also ensure quality. In case of doubt, if these are not processed properly, he must escalate it quickly and find a solution there. At the same time, because it is very time-consuming, this also means that he must have a certain leadership ability in the sense that he is also respected as a project manager.
16:27	Without a leader and without leadership, an M&A team is worth nothing.
3:50	As a manager, clear team leadership and clear distribution of tasks are important.
15:21	Yes, but then also a certain leadership competence. Of course, tasks must also be clearly distributed, not necessarily by the boss, but simply that the tasks within the team are clearly distributed.
Expert	
10:8	B there is a signal effect for companies that these profiles or these applicants also have the appropriate expertise.
16:6	Here I would like to add however that if it concerns an application, nevertheless very well an additional professional examination can be very helpful so to speak, a CVA, a CFA or a WP, in addition, a tax advisor. Due to the fact that you have learned knowledge and know-how in detail, and theoretically technically learned, what you can implement in practice and also enrich your work with a depth, as you could not do without the tool, speaks without the hard skills that someone has previously acquired. The same is true for DDs.
Executor	
3:38	However, I would say that the expertise and guidance of senior colleagues is even more important, as they can theoretically also do the operational work.
Communication	
3:22	Also, communication with the different buyers or with different sellers and also with the own client is important. In the communication among themselves should of course be as optimal as possible. Communication skills would of course be an advantage for this.
5:14	I think a certain level of communication is important. That will come through. I think that’s generally important in the job, that you communicate openly, clearly and in a goal-oriented way.
Relationship Management	
18:18	I think that with every project, regardless of how I’ve experienced it in our company, that with every project the people grow together, that you know each other even better. Of course, there is always friction during the M&A process, which is of course very stressful, but I think that the relationships usually become even deeper.
28:15	So basically, I think the more intense the project is, the more there is a team dynamic. That does not have anything to do with the time span, how long the project runs, so I think a short project or short M&A phase can have just as much development in the team as a very long project. I think at the beginning it’s always a feeling out and then at the end I think the team is welded together in such a way that it can do something best and achieve success there.
19:18	This also creates a bond during the course of a transaction, because everyone wants to complete the transaction.
Negotiation	
20:11	Perhaps also a little willingness to compromise, because at the end of the day a middle ground probably has to be found, and of course it is then always good if you are also prepared to perhaps compromise a little and not always represent your opinion too much, but can also find a good middle ground in a certain way.
23:8	I think it is also very important in the acquisition phase that you have the experience, the empathy, and the willingness to meet the customer halfway.
24:14	Second aspect with regard to the negotiation guidance goes. Here, too, it’s about taking the opposite position to the negotiating partner and influencing the transaction price in his direction. This means taking rather hard positions or building up counter-positions, i.e., thinking very economically, calculating everything precisely and always with a view to the risk.
7:20	To the procedure, to the process, to how the parties behave, with which possibly negotiation tactics you overstimulate situations or not. It’s always a fine line with treatments. You just cannot overdo what you are trying to get, what you want to get and have to make certain concessions. I think it’s easier when you have done it several times. Then you have learned how it works and what makes people tick.
Emotional Intelligence	
13:14	To do this, you need emotional intelligence, and you have to take your employees with you. You have to positively encourage them in this process, that I also accompany this process positively and I have to deal very sensitively with the difference in cultures.
Industrial Knowledge	
25:2	You will not be able to integrate an acquired robotics company into Daimler without having specialists in robotics engineering. You can see the same for banks or other industries.
25:3	I do not think you necessarily need industry expertise for that, but it’s massively helpful to have that. Because you just have to understand very quickly sometimes what world the other people are sitting in. If I have no idea how they talk in the bank, then it’s going to be difficult.
Financial Expertise	
3:8	In my opinion, it is not important that all members of the M&A team are absolute corporate finance experts. But it is definitely important that there are people who are fit in the various corporate finance areas and, accordingly, probably either have the relevant certificates or have studied classical business administration.
M&A Phases and Deadlines	
1:51	At the beginning of the project you are relaxed and confident. Yes, and with the deadline and time pressure, it becomes a bit more stressful and not so relaxed anymore.
3:42	But with some people, you notice that when things get a little more stressful, the manager might put a little more pressure on them.
3:43	The atmosphere is a little less relaxed and you can already tell that the deadline is now influencing the dynamics, because you know that we have to have it ready by the end of the week and it’s no longer this relaxed way.
4:30	I think you work together more efficiently, even if you are a bit annoying to each other. In the course of the project, you simply have the experience, expectation and what it comes down to.
4:31	I would say that the tone is always a little rougher at the end. But the cooperation is already a bit ground in and even if then interpersonally then the other does not fit, you know what is expected approximately.
Shared Language	
1:22	On him it was not, but he should a completely foreign team not only that we were foreign, also still by the language and because he came from Belgium, what else knew, he could not coordinate the whole thing so well
23:6	On the other hand, if you are involved in the transaction I was once involved in with a medium-sized company, you might have a similar appearance. When you are involved in a transaction, it’s more important that you have a similar demeanor. That you speak the same language, that you do not come across as too snooty, that you do not drive up there in a big car.
Relationship and Trust	
21:15	Trust is very important. I notice that sometimes when I work with interns. I think that a good example. That you also pass on work packages to them and that maybe you trust some interns more. If you cannot trust one, then it’s actually more double work that you do.
1:43	Above all, I think it’s important to be trustworthy, to give someone the feeling: Hey, we’ll do it.
2:27	Since we see this as recurring business and also want to build a foundation of trust, I would say it already goes both ways.

### M&A deal outcome

When speaking to M&A professionals from investment banks, consulting firms or corporates, a successful M&A transaction was either defined by its status of completion or by the characteristics of the deal execution. Thus, when elaborating on team roles, soft/hard skills, project timing and team culture, these two conceptualizations need to be kept in mind and other outcomes, such as financial KPIs or employee attitudes move into the background.

Most of the interviewees defined M&A deal outcomes by means of deal completion. Working on M&A transactions is a complex task often including time pressure. Thus, the professionals stated that deals were even more successful, when they were characterized as large and complex projects, as indicated by the following statement:

“Why was this the most successful transaction? Simply for the reason that it was a very complex transaction and the prospects of success were relatively low and to have made the deal in the end was then pretty cool and accordingly also the most successful transaction.” (Interviewee 10)

Besides completing a transaction, professionals noted that a short period to close a deal is an important indicator for a successful M&A transaction. In contrast to the result of a done deal, for many M&A professionals executing a transaction smoothly (without further complications) is an important success indicator.

The execution prior to closing an M&A deal is also of relevance to professionals when evaluating M&A success. The interviewees distinguished mainly between a structured work approach, stakeholder relationships, and team spirit. The latter is described in the following statement:

“That means that across different languages, across different continents, it all worked great as a team and you just felt the spirit, but also got to know different approaches in teams and were able to adapt.” (Interviewee 15)

Interpreting the various statements, we conclude that the interviewees long for structured and smooth M&A deal executions including a strong team spirit, clear separation of work streams and responsibilities, and satisfied stakeholders. A completed transaction might not be perceived as successful, if one of these conditions is not sufficiently met.

### M&A team roles

When it comes to team roles, M&A professionals mostly named project-related team roles. M&A project-related team roles, including the role of project manager, expert and executor, strongly contribute to M&A deal outcomes. Most interviewees noted that team members can only fulfill one of the three roles and consensus was reached that each role is necessary for completing and executing a M&A transaction, thus driving its success.

The role which has been elaborated on the most during the interviews was the *project manager*, who is responsible of keeping a bird’s eye view and allocates tasks among the M&A team. This role can be compared with a coordinator, as the following quote indicates:

“Clearly, first and foremost is the project manager. The project manager must coordinate all workstreams and have a rough overview of the direction of the project. He or she pulls the strings.” (Interviewee 10)

Other interviewees noted that the project manager’s requirement profile looks somewhat different compared to the rest of the team. His/her main responsibility is coordinating the internal M&A team but also being a contact person to other stakeholders who are involved, such as clients, other advisors, and members of the target firm. Thus, communication is one of the most required soft skills in fulfilling this team role. Moreover, a project manager does not have to be deeply involved in all technical matters. Finally, it was stated that an M&A team is worth nothing without a leader. Two other interviewees noted that a project manager’s leadership mostly includes the competence to clearly distribute tasks and delegate responsibilities.

In contrast to the project manager, every M&A team includes members fulfilling the *expert* role. Some of the analyses in M&A projects can only be performed by experts with deep knowledge in a specific domain. One respondent underlines this claim with regard to the topic of target valuation:

“A valuation specialist. This does not mean that he can only do valuation. He can also do other things, but the role itself is always recurring. You always need a dedicated valuation specialist. This person must have extensive financial modeling knowledge and must calculate sensitivities.” (Interviewee 4)

The expert role has a different requirement profile compared to a project manager. For example, his/her externally recognizable hard skills need to be visible to other stakeholders in order to signal confidence and technical correctness. Thus, professional exams and certifications are looked for when staffing an expert to the team. It takes experience and refined soft skills for fulfilling the requirements of the project management or expert role. Juniors in M&A teams often start building up these capabilities by first fulfilling the role of an *executor*. The consequent quote is in line with the interpretation that experts and project managers rather delegate assignments and take decisions when juniors work on the execution: “The regular consultants should then do most of the operational work.” (Interviewee 3). One professional indicated that the project manager and expert role are somewhat more critical, as both could as well execute the operational work if there was no resource restraint. But although executors might be easier to replace, the work needs to be done and this can only be accomplished by involving all three project-related roles.

### Soft skills

When speaking to the participants about how soft skills in M&A teams drive deal completion or its execution, we identified four relevant concepts. These are communication, relationship management, negotiation and emotional intelligence. Interviewees describe communication in its different facets regarding quantity, quality, and direction. One professional describes:

“In any case, communication skills are essential, because, on the one hand you have to get along well with the clients and you have to be able to lead them well, explain a lot to them technically, and on the other hand, you are in constant exchange with potential investors, be it strategists or financial investors, and have to find out their needs.” (Interviewee 14)

The above statement underlines the necessity to provide technical explanations to stakeholders involved. Furthermore, it touches on the direction of communication. Multiple interviewees refer to the importance of clear communication towards all parties. Other professionals define good communication skills by openness, clarity, and goal-orientation.

Another highly discussed soft skill is relationship management. As many stakeholders are involved in a deal and the M&A industry is rather small, it is vital to maintain good stakeholder relationships. One of the professionals, an M&A advisor, elaborated on the importance of maintaining a good client relationship:

“On the one hand, if you already know the customer for a long time and have delivered good work in the past, the trust is built up due to the past work performance and thus you have a good customer loyalty. When you first contact a customer, I think it’s important to listen to the customer, to take on board his points and to try to make him understand and take on board his issues. In other words, you have to give them the feeling that their issues are being addressed and evaluated appropriately.” (Interviewee 28)

Not only the relationship to external stakeholders appears to be vital. Another success-driving factor is the relationship among the M&A team members. The more projects the team members work on together, the more frictionless the collaboration turns out to be.

Along an M&A process, there are constant negotiations about the pricing of the target company, procedural as well as contractual aspects. In order to complete the deal in favor of one’s own interests, these negotiations bear the risk of preventing a smooth deal execution. Thus, negotiation skills become immensely important to balance all stakeholders’ interests and find compromises. One interviewee links negotiation skills to empathy in the following statement:

“What is extremely important here, from my point of view, is empathy, especially in contract negotiations. That means putting yourself in the shoes of others, understanding what the opposing positions are and what my own positions are. Then, of course, negotiating skills, looking at how can I balance the interests? How can I steer both sides somewhere? Of course, I am mandated by one side, but I simply have to have this instinct.” (Interviewee 30)

Besides the human aspect in negotiations, tactics are relevant as well. Negotiations which result in favorable outcomes need to be approached strategically. Thus, professionals develop counter positions and constantly evaluate the monetary impact of subjects for negotiation.

Empathy, together with themes on curiosity and creativity, forms the concept of emotional intelligence. As M&As often have severe consequences for employees in both the target and buyer company, it bears the risk of employee resistance and ultimately M&A failure. This becomes especially important in the post-merger phase, as the following M&A professional describes:

“I think empathy is also super important, because in a classic post-merger integration it is often the case that if you have employees from both companies, they will not keep all employees from both sides. You have to ensure that the people who then no longer have a future in the company at least make a reasonable handover and hand over their issues and work on the project. I think you also need a lot of empathy and a bit of sensitivity, so that you do not have to push everything through your agenda so strictly.” (Interviewee 7)

While in negotiations it seems to be important to have clear agendas and counter positions, it can turn into a disadvantage when executing them strictly. Sensitivity and empathy help with employee interactions in transition phases during an M&A transaction.

### Hard skills

When speaking about hard skills to interviewees, we identified two strongly prevalent themes: industry knowledge and financial expertise. Even though industry knowledge helps M&A teams to better understand business models and evaluate target firms, in the context of our study it is more expressed as a necessary but not sufficient condition for the execution of a deal, as the following interviewee indicates:

“I do not think you necessarily need industry expertise for that, but it’s massively helpful to have that. Because you just have to understand, very quickly sometimes, what world the other people are living in. If I have no idea how they talk in the bank, then it’s going to be difficult.” (Interviewee 25)

If an M&A team has absolutely no specific industry knowledge and it has to integrate the target firm into a new parent company, which is from a different sector, difficulties will arise. Relevant industry knowledge further helps M&A teams in the pre-acquisition phase to identify target firms or potential consecutive buyers, thus reducing the duration of a transaction, as well as transaction costs.

Financial expertise was another important factor when discussing hard skills. Whether it was acquired by academic or professional education, or experience, some professionals noted that it is one of the main resources of M&A professionals and justification of their right to exist:

“Here, it’s about highlighting the risks of a transaction. If the pre-merger phase is more about opening up opportunities, the second phase is more about highlighting the risks, and in this respect, you need people who work very precisely, who have a high level of financial understanding, who can precisely quantify financial risks from an accounting point of view, can evaluate tax risks and can then also compare the entire risk calculation together with the opportunities.” (Interviewee 25)

The quote above indicates that especially in the deal-making phase, where activities like due diligence and valuation are executed, a high level of financial understanding is needed to quantify financial risks.

### M&A project timing

Aside from the individual soft and hard skills, multiple external factors such as the M&A project timing influence M&A team members working towards deal completion. The interviewees explained that the team dynamics change among the M&A phases, as well as through approaching deadlines and milestone achievement. At the beginning of the M&A, the team members are more relaxed and confident about the deal completion. However, towards deadlines, time pressure increases, and the professionals often become stressed. The following quote underlines the tension towards deadlines:

“This dynamic always occurs in the process. So, I have not yet experienced a single process where calmness is present in the entire process. It’s always stressful, there’s always tension. A lot of money is at stake. The parties involved are somewhere at the limit. If you just look at the overall team now, of course you have professionals who are always running against a deadline.” (Interviewee 9)

Despite the imposed stress on the M&A professionals when approaching deadlines or the end of a project, the interviewees also noted a positive effect. The more advanced and closer you get to the final deadlines, the more advanced and efficient the communication and cooperation is within the team.

Another factor which was raised by the interviewees was being staffed on multiple simultaneous M&A projects. According to one professional, M&A deals deserve full attention, however, sometimes potential attractive transactions arise simultaneously. Because of resource constraints, the professionals need to manage multiple deals in this case, as the following quote indicates:

“I think a project manager can handle two or three projects at the most, but only if he has a very good team under him that can handle the majority of the tasks. If we think of the “Swift” project, for example, the engagement manager could not do anything else at all. I had two other projects going on at the beginning of the project, even though I wasn’t even the leading project manager there, and that did not work at all. People confuse things with each other. The counterpart also has little understanding for it, for example, if you want to make appointments, then you have to tell him there is another meeting on the other project. That is gladly accepted, but if it happens all the time, then it is not understood. That leads to stress for the manager.” (Interviewee 13)

The professional further indicates how important it is to have a functioning team around the project manager. Others have indicated that multiple projects are only possible if team members are able to work rather autarkically rather than needing high coordination effort.

### M&A team culture

A further concept influencing team members when working towards M&A deal completion is the team culture. This concept entails the themes of shared language, relationships and trust. The concept of shared language has multiple facets. One interviewee described it as speaking the same national language. If there are differences in the mother tongue team members speak, coordination becomes more difficult. This is true for both internal and external parties in the M&A team.

Speaking the same language can also be defined in terms of having the same understanding and set of perceptions regarding the M&A transaction. One professional noted that there is a difference between speaking and negotiating with mid-market companies and large multinational companies. As the following quote illustrates, ‘language’ can differ as well when speaking to either financial investors (e.g., private equity investors) or corporate buyers:

“A potential buyer may also be a private equity house, which generally ticks quite differently than [corporates], i.e., is even more aggressive than banks […]. Then you first have to make sure that if you approach them directly, you speak the language they speak.” (Interviewee 6)

Most importantly, team culture is formed through trust and relationships. Trust functions in multiple ways. First, project managers need the confidence that their colleagues will accomplish the task given to them, as the following quote indicates:

“Trust, when you mention it, is very important. I have to trust that my colleague, when he gets a task from me, will also carry it out. You can compare it a bit to a team sport. You can see this with colleagues who have also played a team sport. If someone knows that he has a certain role and then gives everything for it and also commits himself to this position, then I think that is extremely important for the success of the entire transaction.” (Interviewee 29)

It is of great importance to trust in the ability of the team members to accomplish their tasks as it makes delegation easier and the amount of work will decrease substantially. Furthermore, it can be a motivator for the executor role, when given the trust and autarky to accomplish tasks themselves. Trust also needs to be established between the potential buyer and seller. As M&A transactions are not necessarily one-time events and business relationships are of a recurring nature, a trustful relationship needs to be established.

With regard to relationships, many interviewed M&A professionals underline the importance of friendship-like relationships within M&A teams. One interviewee describes this as follows:

“Team cohesion is also really important to me. Even though there is of course a separation of professional and private life, I prefer to work with people who are like friends, rather than colleagues, with whom I simply have to spend my time, whether I want to or not. For me, that’s definitely motivating, especially when I’m still working at night during stressful periods. That you still have fun with the people, yes, for me, that would be corporate culture.” (Interviewee 27)

Many others noted that spending a lot of time together and working on multiple M&A transactions make a team grow together and work more efficient. As all team members work towards closing the M&A transaction it creates a bond between them. This is promoting M&A deal outcomes as it is motivating and a driver of communication and coordination.

## Towards a theoretical framework

**Figure 2:**
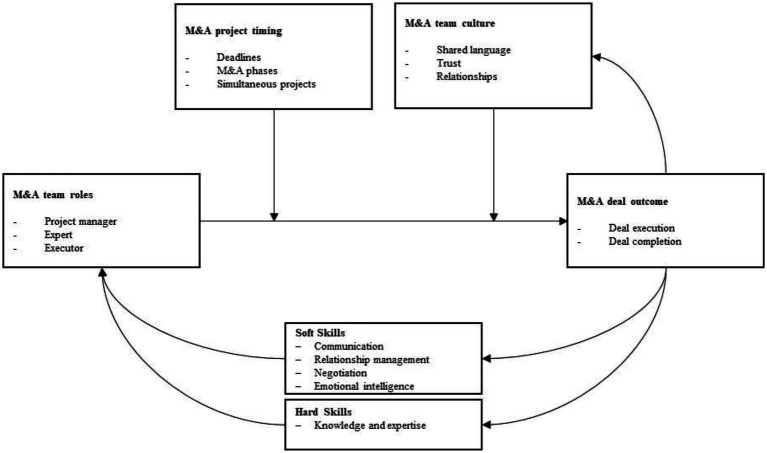
How M&A team roles and skills shape perceptions of M&A deal outcomes.

In this section, we develop an empirically grounded theoretical framework which illustrates how M&A team roles influence M&A deal outcomes and how this relationship is influenced by M&A project timing as well as M&A team culture, as illustrated in Figure 2.

Team roles are crucial for deal completion or a smooth execution. Our data provides insights on the importance of project managers, experts, and executors. All team members are vital in team performance assessments (de Waal et al., 2020), specifically in the context of M&A transactions. As the project managers are responsible for keeping a bird’s eye view and delegation of tasks and responsibilities, they have a coordination and leadership function. Our findings are consistent with prior findings and underline the relationship between project managers and M&A deal outcomes. In this regard, Junni and Sarala (2014) provide an overview of multiple studies indicating how project leader behavior (such as open communication (Saunders et al., 2009) and interpersonal treatment (Klendauer and Deller, 2009)) positively influence merger outcomes. The expert role contributes by having deep knowledge in a specific domain and thus is actively shaping the M&A outcome, as M&A transactions are of large complexity and need experts to be assessed (Gal-Or et al., 2022). Lastly, executors work towards positive M&A deal outcomes by simply executing the operational work. According to Beauchamp et al. (2002), all team roles work equally towards the team’s performance and goals unless role ambiguity exists which would reduce role efficacy.

Our findings support the findings by Jobidon et al. (2017), who argue that role allocation is impacted by the expertise of the different team members which the teams needs to coordinate in order to achieve their goal. We do not only propose that the M&A team roles contribute to its outcome (i.e., M&A deal completion), but we further propose that a manifestation of identified soft or hard skills influences which roles a team member qualifies for at an individual level. As many seasoned professionals develop in either a profound financial expert or a people-skilled project manager throughout their careers, many interviewees connected a set of soft or hard skills with these roles.

In line with the classification of personal, social and methodological soft skills by Heppner (2018), we presented evidence that these skill sets influence team roles and their ability to work towards M&A deal completion. Our data revealed that the emotional intelligence specifically drives interactions with internal and external stakeholders in an M&A context. Similarly, the soft skills communication, relationship management, and negotiation all influence the interactions and interpersonal relationships, as described in previous sections. Therefore, we conclude that the presented soft skills are particularly relevant to project managers working on desired deal outcomes, which is consistent with earlier studies, such as by Rogo et al. (2020) who illustrate that soft skills like relationship management and transformational leadership significantly add to the accomplishment of project goals.

Consistent with the classification of vocational hard skills (Widoyoko, 2009), our data revealed two important hard skills with regard to M&A teams. Industry knowledge and financial expertise are linked in such a way that the more experience professionals in M&A transactions have, the more likely they become aware of industrial or financial specificities. The interpretation of these findings is consistent with Abdullah et al.'s (2018) finding, who argue that employee experience in M&A processes drives M&A success. Of course, industry knowledge and financial expertise can be acquired theoretically. Instead, we argue that by having a mix of junior and senior professionals within the M&A team, junior professionals can leverage the experience of senior peers and thus have greater learning effects with regard to industry knowledge and financial expertise without putting the M&A deal outcome at risk. Our argumentation is in line with Mohammad et al. (2010), who claim that ‘tacit’ (i.e., practical) knowledge acquisition is stronger anchored in the minds of professionals than ‘explicit’ (i.e., theoretical) knowledge acquisition.

We further propose a learning cycle between M&A team members working on M&A transactions and their soft and hard skill development at the individual level. Findings on learning in M&As have been contradicting so far. On the one hand, scholars found evidence of individual CEOs having more negative deal outcomes in second or higher-order deal experience compared to their first deals (Billett and Qian, 2008). Initial positive M&A deal outcomes may drive managerial hubris and overconfidence which endangers the performance of subsequent M&A transactions. In contrast, Collins et al. (2009) found evidence of positive learning effects in M&A transactions due to the application of routines and accumulation of knowledge on, e.g., post-merger integration. Our study elaborates on these findings by illustrating that experience shapes soft and hard skills which consequently optimize the team roles in executing the transaction. In line with this finding, studies in other research contexts have presented evidence that skills are built through repetition (Devroey et al., 2021).

Furthermore, our findings suggest that team members with experience working together on prior M&A transactions have stronger relationships and develop more trust in each other. This is represented by the arrow in the framework pointing from M&A deal outcome to M&A team culture. It shows a positive relationship between deal outcome and the team’s culture, as stronger relationships and trust among team members will be established and the team culture overall becomes more communicative, motivating and friendly when team members are working on multiple projects together. This finding is in line with prior studies underlining the positive impact of team familiarity on team performance (Huckman and Staats, 2011; Staats, 2012).

Moreover, our interviewees underline that a team culture of trust and strong relationships makes the team roles function better when working on the deal. Rezvani et al. (2016) also presented evidence that project managers’ trust towards other team members leads to better project performance and its completion. As trust among team members is an important dimension in shaping team culture (Kane-Urrabazo, 2006), we propose a positive moderating impact of an M&A team culture on the relationship between M&A team roles and desired M&A deal completion. The moderating effect of trust within a team culture on teams and M&A performance has been highlighted by other scholars as well (Weber et al., 2012; Trąpczyński et al., 2018).

Lastly, we need to acknowledge the role of M&A project timing into the proposed theoretical framework. Stress has been found to be of a negative influence in project management (Aitken and Crawford, 2007). As our data illustrates, stress negatively influences M&A teams in executing M&A transactions. It is mostly induced by approaching tight deadlines and reaching the end phase of an M&A project. Multiple projects at the same time further induce all team roles to lose focus and eventually perform worse.

## Discussion and conclusion

With this paper we aimed to explore a composition logic of M&A teams and its consequences for M&A outcomes. We sought to answer the research question: *How do M&A team compositions shape M&A deal outcomes?* Based on 30 interviews with M&A professionals we identified three M&A team roles, which are linked to a set of individual soft and hard skills. The identified team roles influence M&A deal outcomes in the form of deal completion and deal execution by fulfilling their set of responsibilities and tasks, whereas project timing and team culture are important factors influencing this relationship.

### Theoretical implications

Our study has several theoretical implications and paves the way for future research. When looking at the broader context of mergers and acquisitions, we contribute to the rising interest in M&A teams (Aktas et al., 2021). Typical tasks of these teams involve directing transaction rationales and screening potential takeover targets. Effective teams accept deals which are driven by clear strategic rationales and reject those which are driven by cognitive biases, such as managerial overconfidence (Malmendier and Tate, 2008; Aktas et al., 2021). We argue that the M&A team roles have a profound impact on the execution and completion of the respective deal with regard to M&A learning and shaping the skills on an individual level.

As many scholars examined the role of leadership in the context of M&As (Meckl, 2004; Sitkin and Pablo, 2005; Junni and Sarala, 2014), we shifted the focus away from the concentration around a leadership perspective towards a more team-centric view. According to Segal et al. (2021), many scholars shed light on M&A stakeholders with regard to target employees, shareholders, customers, supplies, community, and the government. However, the actual M&A team executing the transaction has been neglected so far. We fill this research gap by examining how these teams are composed and affecting the M&A’s outcomes. In contrast to the on-going discussion about the effects of either national or organizational culture on integrating two merging companies (Warter and Warter, 2017; Galpin, 2019; Siebecker and Lozano, 2020), cultural influences among the single M&A team members are also of interest, as our findings highlight.

A main contribution of our study is the presented learning cycle within M&A teams. On an individual level this could shape the skill sets of team members and thus optimizing the deal execution. Existing literature illustrates the multi-facetedness of M&A learning (Zollo and Meier, 2008). On the one hand, studies on managerial overconfidence demonstrate that learning induces top executives to develop the so-called confirmation bias which causes worse decision-making and eventually value-destruction (Billett and Qian, 2008; Garbuio et al., 2010). Relatedly, Zollo (2009) argues that the M&A managers’ perception of their success deviates more from objective performance measures when accumulating M&A experience. Zollo and Singh (2004) further state that firms with post-merger integration experience do not evidently execute better performing post-merger integrations. On the other hand, studies found positive effects of learning in an organizational context with regard to, e.g., post-merger integration (Collins et al., 2009). We enrich this current body of knowledge by adding insights on positive learning effects at the individual level. We argue that learning in the context of M&A transactions has multiple facets and our findings support the claim that learning could be positive in terms of deal outcomes. Due to the positive effects of M&A learning, Trichterborn et al. (2016) suggest that companies should have dedicated M&A functions which will accumulate deal experience and therefore drive deal outcomes.

We further evaluated M&A team internal capacities with a focus on skill sets and team roles. On the basis of our study, we now better understand which skill sets and team roles are beneficial when executing an M&A transaction, however, it remains unknown which project management approaches and task completion measures are helpful. With rising regulation in the M&A market, Campbell et al. (2021) argue that M&A teams need to apply more agile project measures to successfully clear hurdles. However, agile project measures largely depend on the stakeholder’s experience with it. Our findings point to the role of organizational culture which lays the foundation of whether agile project management works out well or induces dissatisfaction among team members. Still, further investigation and research needs to be done on how transformations, such as digitization, can be successfully overcome with regard to project management approaches and task completion.

### Practical implications

As our data suggest a differentiation of roles within M&A teams into project managers, experts, or executors, we suggest M&A leaders to reflect on their team and categorize their members accordingly. Furthermore, soft and hard skills of each employee need to be evaluated. In this way, leaders will be able to unfold a clearer picture of their existing team constellation. Role-specific task assignment could be a step towards employees working more efficiently and eventually becoming more satisfied matching their set of skills and team role.

Moreover, given the identified skill set, personal development can be pursued on the job. Each M&A professional might assess his or her own skill set and actively work on enhancing the identified soft skills, for example. At the same time, motivation and willingness to grow forms the basis for enhancing both soft and hard skills.

Furthermore, M&A leaders can include the illustrated soft and hard skills as criteria into their decision-making on M&A team composition. When looking for new hires, they would need to interview the candidates accordingly in order to make the right decisions, for example in relation to emotional intelligence.

Lastly, we suggest corporate management to critically evaluate how many M&A deals managers and employees are staffed on, as multiple simultaneous projects and tight deadlines lead to stress which affects their focus negatively. As our study suggests, room for establishing relationships and evolving trust forms the basis for efficient teamwork and thus could be offered to M&A teams through, e.g., team events and joint off sights.

### Limitations and future research opportunities

With the attempt to generate and develop theory, one must be careful to generalize the presented findings to other contexts. Generalizability has been discussed controversially with regard to qualitative studies (Carminati, 2018). Following an interpretivist research philosophy, we try to focus on providing in-depth explanations and meanings as well as the exploration of new concepts rather than generalizing our findings. Especially, as we have interviewed only professionals from German-speaking countries, the results derived from the interviews may vary in different geographic areas. Furthermore, our sample included only 20% of women. According to Affleck et al. (2013) interviewing an unbalanced set of men and women might distort the findings, as men tend to have a lack of emotional expression. However, we justified interviewing a gender unbalanced sample, because of the low concentration of women working in finance (Sahay and Cihak, 2018). Based on these limitations, future research could be pointed towards different research contexts including interviewees from other nations.

As team culture is a part of our presented findings, other national cultures might lead to different views with regard to, for example, friendship-like relationships or trust. Compared to Western countries, where individualism is more prevalent, in Asian countries teamwork and a collective understanding contribute even more to M&A transactions (Cohen et al., 2016).

With regard to our qualitative research approach, another limitation is related to result verification as the participants are mostly in control over the data collected and the researchers are limited to objectively verifying the results (Morse, 2001). As stated in this paper’s methodology section, we tried to reduce this limitation by implementing measures to increase trustworthiness, related to the credibility, dependability, transferability, and confirmability of our study (Lincoln and Guba, 1985).

The chosen medium for interviewing the M&A professionals was the voice-over-IP video software Zoom. Even though there is a small risk of non-verbal information being lost, scholars legitimize the use of these videotelephony software and point out the advantage of being able to interview a more varied sample (Lo Iacono et al., 2016). In line with this, an interesting new research stream discusses the M&A teams’ capability to successfully complete a transaction in light of increasing digitization (Kotarba, 2018). This on-going transformation process induces many challenges which interfere with the entire organization (Ikegami and Iijima, 2020). Although digitization offers the potential of more efficiency, it bears risks and challenges for M&As, as well. M&A teams need to acquire an understanding of the digital gap between acquirer and target. The implementation and interpretation of new key performance indicators could be a starting point (Kotarba, 2018). Thus, a future focus on team transformations and digitization in an M&A context is a longed-for research area.

## Data availability statement

The raw data supporting the conclusions of this article will be made available by the authors, without undue reservation.

## Ethics statement

This study involving human participants was reviewed and approved by the Ethical Review Board at Vrije Universiteit Amsterdam. The participants provided their written informed consent to participate in this study.

## Author contributions

TP, DK, and SK contributed to conception and design of the study and wrote sections of the manuscript. TP organized the database, collected the data, and wrote the first draft of the manuscript. All authors contributed to manuscript revision, read, and approved the submitted version.

## Conflict of interest

The authors declare that the research was conducted in the absence of any commercial or financial relationships that could be construed as a potential conflict of interest.

## Publisher’s note

All claims expressed in this article are solely those of the authors and do not necessarily represent those of their affiliated organizations, or those of the publisher, the editors and the reviewers. Any product that may be evaluated in this article, or claim that may be made by its manufacturer, is not guaranteed or endorsed by the publisher.

## Appendix I – Interview protocol

**Table:**

Introducing Narrative Questions	
I	Please tell me about the most successful investment you have been working on from acquisition to divestment. How was the outcome related to the M&A team executing the transaction?
II	Please tell me about the worst performing investment you have been working on from acquisition to divestment. How was the failure related to the M&A team executing the transaction?
**Hard Skills Questions**	
I	How would you compose an optimal M&A team with regard to hard skills?
II	Within the pre-merger phase, when evaluating industries or targets to invest in, which hard skills are required mostly and why?
III	Within the acquisition phase, when due diligences and company valuations are performed, which hard skills are required mostly and why?
IV	Within the post-merger phase, integrating the merged firms and value creation is pursued, which hard skills are required mostly and why?
**Soft Skills Questions**	
I	Within the pre-merger phase, when evaluating industries or targets to invest in, which soft skills are required mostly and why?
II	Within the acquisition phase, when negotiations are held and contracts established, which soft skills are required mostly and why?
III	Within the post-merger phase, integrating the merged firms and value creation is pursued, which soft skills are required mostly and why?
**Team Roles Questions**	
I	Which team roles do you perceive as dominant and recurring in M&A projects and why?
II	How do certain team roles or constellations stimulate the success or failure of an M&A transaction?
III	Should an M&A team structure be different among the phases pre-merger, acquisition and post-merger? If yes, why?
IV	How do team dynamics evolve around M&A projects?
V	Are there any conflicts between the acquiring and acquired firm and how should they be dealt with?
**Concluding Questions**	
I	Do you work in an optimal composed M&A team? If there is room for improvement, how can team members adapt or acquire skills on the job?
II	How does the decision-making process around M&A teams look like in general? Based on what factors are decisions in the M&A team being made?
III	Which other factors such as corporate politics, communication, trust, etc. play a role in M&A teams?
